# Classification of health needs: a cluster analysis of older adults in urban areas

**DOI:** 10.1186/s12877-023-04333-y

**Published:** 2023-10-09

**Authors:** Liu Yang, Quan Zhou, Congzhi Wang, Dongmei Zhang, Ting Yuan, Xiaoping Li, Yunxiao Lei, Lin Zhang

**Affiliations:** 1 School of Nursing, Wannan Medical College, 22 Wenchang West Road, Higher Education Park, 241002 Wuhu City, An Hui Province P.R. China; 2 Department of nursing, Yijishan Hospital, the First Affiliated Hospital of Wannan Medical College, Zheshan West Road, Yijishan District, 241004 Wuhu City, Anhui Province P.R. China

**Keywords:** Aged, Health status, Health services needs and demand, Cluster analysis, Community Health Services

## Abstract

**Background:**

In this study, a cross-sectional survey was used to understand and analyze the health status and health needs of the elderly in the community. The cluster analysis method was used to explore the relationship between health needs items and investigate the commonness among health demand items, to provide a reference for the development of health management of the elderly with chronic diseases.

**Methods:**

We used convenience sampling to recruit the participants (aged 60 and above) from four urban community centers in Jinzhou City, Liaoning Province, China, in this study. This study uses the Medical Outcomes Study(MOS)36-Item Short-Form Health Survey. The self-designed questionnaire includes sociodemographic characteristics, chronic diseases, physical examination conditions, illness in the past two weeks, and a health needs questionnaire. SPSS 18.0 was used for data entry and analysis. Data analysis methods included descriptive statistical analysis, t-test, one-way analysis, cluster analysis, and linear multiple regression analysis.

**Results:**

The rate of health needs among the elderly in the community for various health services is 1.3–69.7%, of which the top three are: regular physical examination (69.7%), day Care Center (67.7%), the establishment of a Medical Alert Systems (66.1%). The health needs of the elderly in the community are divided into three categories: basic needs (24 items), health education (13 items), and first aid (2 items). The regression analysis found that the influencing factors of health status were age, revenue and expenditure, medical expenses, health education, basic needs, and first aid.

**Conclusions:**

The community should strengthen the management of chronic diseases of the elderly and the publicity and education of related knowledge, and provide complementary health care services according to the health needs of the elderly, improve the health of the elderly, and improve the quality of life of the elderly.

**Supplementary Information:**

The online version contains supplementary material available at 10.1186/s12877-023-04333-y.

## Introduction

Population aging is the 21st century’s dominant demographic phenomenon in China. China currently houses the world’s largest population of 1.4 billion (19.13% of the world population) and is rapidly transforming into an aging nation. In 2010, there were 111 million (8.2% of the country’s population) elderly aged 65+; among them, 19.3 million were the oldest-old (aged 80+) [1]. In developing countries, increasing longevity, declining fertility, upturning living standards, and the progression of large-sized cohorts to older ages are causing a continuous increase in the proportion of older people worldwide. These demographic changes are associated with an increase in the prevalence of chronic diseases, which presents a challenge for healthcare systems [2].

The need is a critical concept within the international health system, given its driving force in health care policy, development, and delivery at population and individual levels. Needs assessments are pivotal to ensuring continued demand for health services and identifying new target populations with unmet needs. The concept of need is based on a variety of theoretical definitions from a variety of different disciplines [3].

The World Health Organization’s definition of health is often used: “Health is a state of complete physical, psychological, and social wellbeing and not simply the absence of disease or infirmity.“ Healthcare needs can benefit from health care (health and physical education, disease prevention, diagnosis, treatment, rehabilitation, and palliative care). Most medical staff will consider needs in terms of healthcare services that they can supply [4]. Health needs include social and environmental determinants such as deprivation, housing, diet, education, and employment. This definition allows us to move beyond the confines of the medical model based on health services to the broader implications of health. With the progress of the times, human health needs are constantly changing.

However, the health of the elderly is a problem that cannot be ignored, and current research on health needs is almost all for all age groups or adult groups [5]. Few scholars pay attention to the middle-aged and elderly, a relatively particular group that needs urgent attention. The community is the primary environment for the elderly to live and the central place for the health needs of the elderly. Community health intersects healthcare, economics, and social interaction [6]. How the community provides standardized service items that meet the health needs of the elderly so that they can receive better community medical, nursing, and rehabilitation services is a hot issue in the current community health research on the elderly.

This study adopted the cross-sectional survey method to understand and analyze the health needs of the elderly in the community. Cluster analysis is a statistical method for processing data. It organizes items into groups or clusters based on their closely associated characteristics. Cluster analysis, like reduced space analysis (factor analysis), is concerned with data matrices in which the variables have not been partitioned beforehand into criterion versus predictor subsets. Cluster analysis was used to explore the relationship among health needs items and analyze the commonness among health needs items, to provide scientific reference for health management, health evaluation, and health intervention of the elderly in the community.

## Materials and methods

### Study population and data collection

From March to June 2011, older adults aged 60 and above from four urban community centers in Jinzhou City, Liaoning Province, China, were approached to participate in this study. Jinzhou has been a communication hub linking northeast and north China ever since. 2020 data shows the total population was 2.893 million, with approximately 830,000 people aged 60 and above. Based on the criterion proposed by Kendall (10 to 20), fold the number of items, and expand by at least 10% to ensure a sufficient sample size. A sample size of at least 396 was calculated since the number of items in SF-36 is 36 [7]. In these four community centers, 642 participants were willing to participate in the study. Participants aged 60 and above, who have lived in the community for a long time, can complete the body assessment test and are willing to participate in this survey. People who did not have basic language communication skills and critical illness were excluded. In total, 611 (95.2%) participants completed the questionnaire.

### Questionnaire

This study uses the Medical Outcomes Study(MOS)36- Item Short-Form Health Survey (SF-36) [8, 9], which was specifically designed for use in the general population and among ambulatory patients. It is widely used in various and with reasonable confidence and effectiveness. The SF-36 has been tested in 260 clinical settings and translated into ten foreign languages. It is a practical and valid instrument for measuring health status and outcomes from the patient’s viewpoint.

The Chinese version of the SF-36 consists of includes one multi-item scale that assesses eight health concepts: physical functioning (PF), role physical (RP), bodily pain (BP), general health (GH), vitality (VT), social functioning (SF), role emotional (RE), mental health (MH), reported health transition (HT). PF, RP, BP, and GH are physiological health, VT, SF, RE, and MH are mental health, and the higher the score, the better the health condition.

In addition, the self-designed questionnaire includes sociodemographic characteristics, chronic diseases, physical examination conditions, illness in the past two weeks, and a health needs questionnaire [10, 11]. We used a 4-point Likert scoring method, with 1 = no need, 2 = low need, 3 = moderate need, and 4 = high need. Participants were categorized based on their state of health needs, with “no need” and “low need” as the “no health need group,“ “moderate need,“ and “high need” as the “health need group.“ The test-retest reliability is 0.861, the internal consistency reliability of this survey is 0.821, and the factor analysis shows that the structural validity is 0.76. This questionnaire has good reliability and validity.

### Data entry and statistical analysis

Data were entered using Epidata version 3.1, which can limit the input outliers. Two researchers worked independently to enter and check the data. SPSS version 18.0 was used for data analysis. A two-tailed probability level of *p*<0.05 was chosen as the level of statistical significance. Missing data were excluded from the analysis. Descriptive statistics were used to describe study participants’ characteristics, the score of SF-36 and health needs. A t-test was used to compare the effect of gender on health status. One-way analysis of variance was used to compare the effects of participants’ characteristics such as age, educational levels, and Marital status on the health status of the elderly. Cluster analysis adopts systematic cluster analysis, the measurement method between different classes adopts the inter-group connection method, and the distance measurement method adopts the Euclidean distance square method. Stepwise multiple linear regression was used to analyze the influencing factors of health status with the total score of the SF-36 scale as the dependent variable. The three categories of “basic needs,“ “health education,“ and “first aid” are independent variables that were taken as the dependent variable to analyze the influencing factors of health needs.

## Results

### Sociodemographic characteristics

Of 611 participants, the mean ± SD age was 65.53 ± 5.93 years, and the age range was 60–92 years. There was 420 female (68.73%) and 191 male (31.26%) participants in the study group. Of the participants, 91.48% were married, 46.48% were in high school, and 65.14% had medical insurance. Table 1 shows the health status by gender, age group, levels of education, marital status, the situation of revenue and expenditure, and medical expenses. Gender, age, marital status, revenue and expenditure, and medical expenses were significantly associated with health status (*P* < 0.05) (Table 1).

**Table 1:** Baseline characteristics with total samples(‾x±s)

	N (%)	PF	RP	BP	GH	VT	SF	RE	MH	Physical health	Mental health	Total	t/F	*P*
**Gender**														
Male	191(31.26)	72.3 ± 28.98	62.15 ± 44.1	82.9 ± 20.53	61.45 ± 19.05	74.96 ± 26.76	82.86 ± 23.78	64.41 ± 42.54	68.98 ± 19.79	278.79 ± 83.45	291.2 ± 80.34	569.99 ± 150.41	2.801	0.005
Female	420(68.73)	82.7 ± 21.08	70.83 ± 40.51	82.71 ± 20.94	63.7 ± 20.15	76.16 ± 17.64	86 ± 22.45	69.21 ± 41.31	74.28 ± 24.54	299.95 ± 76.58	305.65 ± 76.17	605.6 ± 143.44		
**Age (yr)**														
60–64	327(53.52)	84.76 ± 18.47	74.03 ± 38.96	84.56 ± 19.68	64.03 ± 20.17	76.81 ± 17.02	87.84 ± 21.2	69.69 ± 40.95	73.6 ± 21.84	307.37 ± 73.72	307.93 ± 72.52	615.3 ± 136.23	7.826	< 0.001
65–69	179(29.30)	77.57 ± 26.71	65.68 ± 43.11	82.16 ± 19.43	62.86 ± 20.62	75.03 ± 27.2	84.78 ± 22.37	70.26 ± 41.21	69.77 ± 19.01	288.27 ± 80.56	299.84 ± 79.45	588.12 ± 146.59		
> 70	105(17.18)	71.07 ± 28.12	56.30 ± 44.44	79.37 ± 24.97	60.93 ± 18.32	74.32 ± 19.39	79.57 ± 25.97	56.83 ± 44.07	74.7 ± 30.55	267.67 ± 85.67	285.43 ± 86.36	553.10 ± 163.64		
**Educational levels**														
Elementary school and below	14(2.29)	79.82 ± 21.61	70.23 ± 40.28	80.95 ± 20.29	58.45 ± 20.34	72.89 ± 17.68	81.31 ± 20.7	64.27 ± 41.56	71.62 ± 31.43	289.44 ± 74.09	290.09 ± 78.02	579.53 ± 142.99	0.801	0.494
High school	284(46.48)	80.3 ± 21.04	66.09 ± 41.97	81.24 ± 22.95	64.31 ± 19.45	75.49 ± 17.67	83.47 ± 25.1	65.8 ± 43.12	74.12 ± 23.05	291.93 ± 79.5	298.88 ± 76.93	590.81 ± 147.9		
Junior college	195(31.91)	79.87 ± 25.8	67.93 ± 43.35	83.51 ± 19.28	62.57 ± 20.04	76.08 ± 18.95	85.89 ± 22.18	68.73 ± 40.98	71.97 ± 19.98	293.88 ± 82.67	302.67 ± 78.82	596.55 ± 152.02		
Undergraduate degree and above	118(19.31)	77.63 ± 28.69	71.05 ± 39.99	85.87 ± 20.26	66.24 ± 18.93	78.8 ± 29.79	90.41 ± 20.87	71.93 ± 41.02	72.73 ± 19.53	300.8 ± 78.09	313.86 ± 75.87	614.66 ± 136.84		
**Marital status**														
Single	16(2.62)	73.33 ± 34.68	68.33 ± 40.61	85.6 ± 15.44	66.34 ± 18.56	72.67 ± 15.45	90 ± 26.39	75.56 ± 38.76	72.78 ± 16.88	293.6 ± 83.08	311.00 ± 84.09	604.60 ± 160.12	3.531	0.015
First married	529(86.58)	80.45 ± 23.34	68.89 ± 41.65	83.12 ± 20.40	62.98 ± 20.26	76.28 ± 21.32	85.58 ± 22.77	68.69 ± 41.49	72.18 ± 18.54	295.45 ± 79.17	302.74 ± 76.57	598.18 ± 145.09		
Divorced	36(5.89)	67.78 ± 28.84	58.33 ± 41.46	74.67 ± 27.15	58.56 ± 11.23	68.33 ± 11.99	80.51 ± 25.07	48.48 ± 41.38	61.11 ± 15.02	259.34 ± 77.89	258.43 ± 60.45	517.77 ± 123.70		
Remarried	30(4.91)	78.10 ± 23.58	65.44 ± 43.53	77.77 ± 25.57	64.21 ± 17.45	76.09 ± 18.94	76.78 ± 25.19	64.71 ± 42.59	78.46 ± 51.92	285.52 ± 79.77	296.03 ± 96.06	581.55 ± 166.16		
**The situation of revenue and expenditure**														
Income surplus	362(59.24)	79.55 ± 24.23	69.98 ± 40.51	85.56 ± 19.36	63.33 ± 18.84	78.64 ± 24.06	89.09 ± 21.43	69.12 ± 41.34	73.38 ± 17.80	298.42 ± 76.92	310.22 ± 73.25	608.64 ± 137.49	18.220	< 0.001
Balance of payments	29(4.75)	80 ± 22.66	65.61 ± 42.48	81.68 ± 21.41	63.22 ± 20.47	74.44 ± 18.28	84.07 ± 23.42	66.25 ± 42.04	71.33 ± 18.46	290.50 ± 79.18	296.09 ± 77.72	586.60 ± 147.62		
Expenditure exceeds income	220(36.01)	64.66 ± 36.13	61.21 ± 45.6	76.14 ± 24.25	56.59 ± 19.32	70.07 ± 20.06	75.35 ± 23.22	63.22 ± 43.96	65.55 ± 20.41	258.59 ± 93.43	274.18 ± 79.28	532.78 ± 161.68		
**Medical expenses**														
Free medical service	33(5.41)	78.29 ± 24.08	65.85 ± 42.93	84.66 ± 18.8	65.4 ± 19.23	76.52 ± 17.34	85.65 ± 21.55	66.18 ± 42.33	72.76 ± 27.29	294.21 ± 78.52	301.11 ± 78.09	595.32 ± 146.69	7.820	< 0.001
Medical insurance	398(65.14)	80.51 ± 23.74	68.89 ± 41.09	81.69 ± 22.1	61.69 ± 19.82	75.59 ± 23.19	85.36 ± 23.62	68.75 ± 41.15	72.98 ± 17.74	292.78 ± 80.96	302.68 ± 78.48	595.46 ± 148.13		
Self-financed medical service	180(29.46)	60 ± 38.51	75 ± 50	81.5 ± 27.39	46.5 ± 9.54	73.75 ± 13.15	96.76 ± 12.09	41.67 ± 50	68.75 ± 14.63	263 ± 72.5	280.93 ± 71.85	543.93 ± 143.79		

Physical functioning (PF), role physical (RP), bodily pain (BP), general health (GH), vitality (VT), social functioning (SF), role emotional (RE), mental health (MH),PF, RP, BP, GH is physiological health, VT, SF, RE, MH is mental health

### The distribution of health demand indicators for the elderly in the community

The health needs scores of the elderly in the community are shown in Table 2. After categorizing “no need” and “low need” as “no health need group,“ “moderate need,“ and “high need” as “health need group,“ the distribution of health needs of the elderly in the community is 51.62–68.38%. The top three are regularly organized physical examinations (69.72%), Day Care Centers (67.41%), and establishing Medical Alert Systems (66.23%). The bottom three are catheterization (1.3%), regular door-to-door service (2.0%), and nasogastric feeding (2.1%).

**Table 2:** Scores of health needs and need rates of community elders(n = 611)

Number	Health Needs Project	Score (‾x±s)	Health Need Rate (%)
1	Lecture on mental health	1.34 ± 1.22	266(43.5)
2	Rehabilitation exercise knowledge	1.59 ± 1.19	315(51.6)
3	Dietary health knowledge	1.62 ± 1.18	321(52.5)
4	Knowledge of medication safety	1.63 ± 1.17	323(52.9)
5	Knowledge of chronic health care	1.55 ± 1.18	307(50.2)
6	Knowledge of common infectious disease prevention	1.62 ± 1.17	321(52.5)
7	Catharsis on the use of rehabilitation training and rehabilitation physiotherapy instruments	1.67 ± 1.18	331(54.2)
8	Family first aid measures for cerebral hemorrhage	1.53 ± 1.19	303(49.6)
9	Knowledge of family rescue of hyperglycemia crisis or hypoglycemia coma caused by diabetes	1.51 ± 1.19	299(49.0)
10	First aid knowledge for the timely management of traumatic injuries in the fractured limb	1.56 ± 1.19	309(50.6)
11	All-day telephone consultation service	1.62 ± 1.70	321(52.5)
12	Community Medical Care Welfare and Resource Introduction Center	0.21 ± 0.58	42(6.9)
13	First aid knowledge of poisoning	0.20 ± 0.40	40(6.5)
14	First aid measures for upper gastrointestinal bleeding	0.12 ± 0.33	24(3.9)
15	Day Care Centers	2.07 ± 1.96	412(67.4)
16	Oxygen inhalation	1.80 ± 2.22	357(58.4)
17	Increase the construction of facilities and places for elderly fitness and entertainment activities in the community	2.00 ± 1.98	397(65.0)
18	Regularly organized physical examinations	2.15 ± 1.54	426(69.7)
19	Blood glucose monitoring	1.98 ± 2.05	392(64.2)
20	Chronic disease case management / health card establishment	1.79 ± 2.24	355(58.1)
21	Electrocardiogram	1.90 ± 2.53	376(61.5)
22	Hospice care	1.97 ± 1.32	389(63.7)
23	Community geriatric care specialist clinic	1.87 ± 1.86	370(60.6)
24	Intramuscular and intravenous injection	1.92 ± 1.64	381(62.4)
25	Emergency care for sudden illness	1.56 ± 0.81	309(50.6)
26	Establish a Medical Alert Systems	2.04 ± 0.76	404(66.1)
27	Physical cooling	0.27 ± 0.61	54(8.8)
28	Wound dressing change washing	0.32 ± 0.86	64(10.5)
29	Family bed	0.21 ± 0.58	42(6.9)
30	Auxiliary expectoration	0.43 ± 0.65	85(13.9)
31	Help relieve pain	0.26 ± 0.92	51(8.3)
32	Oral health care	0.53 ± 0.87	105(17.2)
33	Specimen collection	0.15 ± 0.68	30(4.9)
34	Enema	0.06 ± 0.24	13(2.1)
35	Nasogastric feeding	0.14 ± 0.35	27(4.4)
36	Auxiliary defecation	0.17 ± 0.56	34(5.6)
37	Urinary incontinence bladder training guidance	0.15 ± 0.36	30(4.9)
38	Regular door-to-door service	0.06 ± 0.47	12(2.0)
39	Catheterization	0.04 ± 0.19	8(1.3)

1 = no need, 2 = low need, 3 = moderate need, and 4 = high need

With the total score of SF-36 as the dependent variable and the three categories of “basic needs,“ “health education,“ and “first aid” as the independent variables, the linear multiple stepwise regression method is used to establish the equation model (Table 3). Age, revenue and expenditure, medical expenses, health education, basic needs, and first aid were the independent factors affecting health status.

**Table 3:** Significant factors associated with SF-36

Variable	Unstandardized B	Coefficients Std. Error	Standardized Coefficients Beta	t	P	95.0% Confidence Interval for B	
						Lower Bound	Upper Bound
(Constant)	6.015	0.412		14.595	0.000	5.206	6.825
Age	-0.306	0.074	-0.168	-4.142	0.000	-0.451	-0.161
The situation of revenue and expenditure	-0.247	0.091	-0.113	-2.706	0.007	-0.426	-0.068
Medical expenses	0.214	0.105	0.085	2.039	0.042	0.008	0.419
First aid	-0.019	0.001	-0.537	-28.105	0.000	-0.021	-0.018
Basic needs	-0.026	0.001	-0.351	-19.198	0.000	-0.028	-0.023
Health education	-0.018	0.001	-0.276	-15.025	0.000	-0.020	-0.016

The total score of SF-36 was used as the dependent variable,and the three categories of “basic needs”, “health education” and “first aid” are independent variables

Gender (Male = 1, Female = 2), age, educational levels (elementary school and below = 1, high school = 2, junior college = 3, undergraduate degree and above = 4), marital status (divorced = 1, single = 2, remarried = 3, first married = 4), the situation of revenue and expenditure(income surplus = 1, balance of payments = 2, expenditure exceeds income = 3), medical expenses (self-financed medical service = 1, medical insurance = 2, free medical service = 3)

### Cluster analysis of the health needs of the elderly in the community

**Fig. 1:**
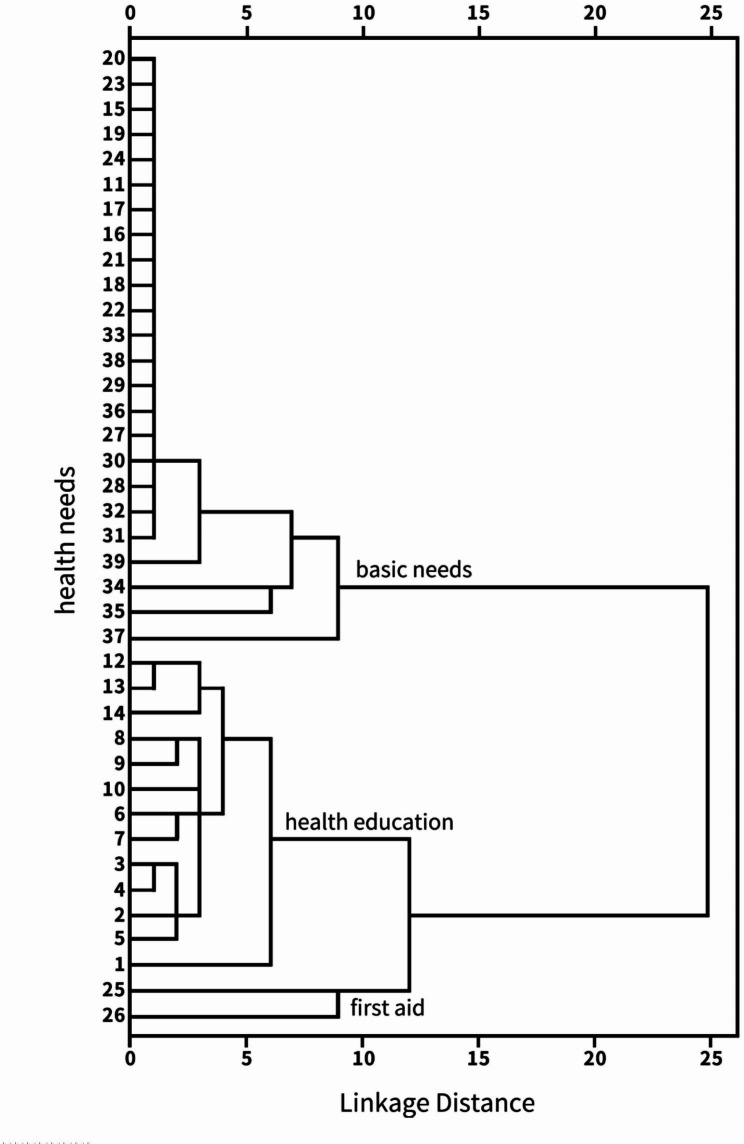
Tree diagram of cluster analysis of health needs of community elders

A hierarchical clustering method was applied to identify groups with similar characteristics of health needs among older adults. A squared Euclidian distance metric was used to measure the distance between clusters on the items of the health needs questionnaire, and Ward’s minimum variance method was employed as the clustering method. With all metrics, three clusters were selected from the resulting dendrogram (Fig. 1).

The indicators of health needs were separated into three categories. The first category was labelled as “basic needs.“ This category consisted of 24 items: chronic disease case management, health care establishment, community geriatric care specialist clinic, Day Care Centers, blood glucose monitoring, Intramuscular, and intravenous injection, All-day telephone consultation service, increase the construction of facilities and places for elderly fitness and entertainment activities in the community, oxygen inhalation, electrocardiogram, periodic tissue examination, hospice care, specimen collection, regular door-to-door service, family bed, auxiliary defecation, physical cooling, auxiliary expectoration, wound dressing change washing, oral health care, help relieve pain, catheterization, enema, nasogastric feeding, urinary incontinence bladder training guidance. Items with the highest scores were periodic tissue examinations. They increased the construction of facilities and places for elderly fitness and entertainment activities in the community for the elderly.

The second category was labelled as “health education.“ This category included 13 items: Community Medical Care Welfare and Resource Introduction Center, knowledge of common infectious disease prevention, dietary health knowledge, Knowledge of medication safety, rehabilitation exercise knowledge, knowledge of chronic health care, lecture on mental health, catharsis on the use of rehabilitation training and rehabilitation physiotherapy instruments, first aid measures for upper gastrointestinal bleeding, family first aid measures for cerebral hemorrhage, knowledge of family rescue of hyperglycemia crisis or hypoglycemia coma caused by diabetes, first aid knowledge for the timely management of traumatic injuries in the fractured limb, first aid knowledge of poisoning. Items with the highest scores were catharsis on using rehabilitation training and rehabilitation physiotherapy instruments, knowledge of medication safety, and Dietary health knowledge. The last category, “first aid,“ involved establishing Medical Alert Systems and emergency care for sudden illness.

### Analysis of the factors influencing the health status of the elderly

The total score of SF-36 was used as the dependent variable, and the three categories of “basic needs,“ “health education,“ and “first aid” are independent variables. The assignment of independent variables: Gender (Male = 1, Female = 2), age, educational levels (elementary school and below = 1, high school = 2, junior college = 3, undergraduate degree and above = 4), marital status (divorced = 1, single = 2, remarried = 3, first married = 4), the situation of revenue and expenditure (income surplus = 1, balance of payments = 2, expenditure exceeds income = 3), medical expenses (self-financed medical service = 1, medical insurance = 2, free medical service = 3). Stepwise multiple linear regression was used to analyze the influencing factors of health status with the total score of the SF-36 scale as the dependent variable (Table 3). The regression analysis found that the influencing factors of health status were age, revenue and expenditure, medical expenses, health education, basic needs, and first aid.

## Discussion

The Constitution of the World Health Organization (WHO) states that “enjoyment of the highest attainable standard of health is one of the fundamental rights of every human being without distinction of race, religion, political belief, economic or social condition” [12]. For a long period of human history, everyone has wanted to be healthy, which is just a kind of human desire. In modern society, health has become a prevalent idea and is considered a right for everyone. The life expectancy of the elderly population is closely related to healthy aging. Poor health harms the elderly and the whole of society. However, because of material conditions, chronic diseases such as hypertension, stroke, and other cardiovascular diseases have seriously affected the health of Chinese residents [13]. The results of this study show gender, age, marital status, the situation of revenue and expenditure, and medical expenses were significantly associated with health status. The study used linear correlation and stepwise regression methods. The results showed that revenue and expenditure, medical expenses, health education, basic needs and first aid were considered independent factors affecting health status.

This survey shows that with the increase in age, the physical and mental health of the elderly is declining. Biologically speaking, normal aging is a spontaneous and inevitable process of organisms over time. It is a complex natural phenomenon that manifests in degenerative changes in structures and the decline of functions, with diminished adaptability and resistance [14]. Research has documented many physiological and mental health benefits of social support, including improved immune, cardiovascular, and neuroendocrine function; positive adjustment to chronic disease; decreased depression and anxiety; and effective buffering against the harmful effects of stress [15]. The other study shows that this may be related to women being more attentive to their feelings, being more sensitive to their physical conditions and feeling more unwell, and using more health service resources than men. According to the National Center for Statistics, women live longer than men and use more health services than men. Therefore, women have more opportunities for early detection, diagnosis and treatment of their own diseases [16]. The general improvement of residents’ health status is one of the main signs of social development and progress. There is always a gap in people’s health conditions in any society, which is primarily closely linked to differences in socioeconomic status. This study found significant differences in health and psychology among the elderly with different incomes [17].

In this study, 32.43% of the elderly suffer from a single chronic disease, and 67.47% of the elderly suffer from multiple chronic diseases. Nearly seven-tenths of older adults suffer from hypertension, cerebrovascular disease, diabetes, ischemic heart disease, or chronic obstructive pulmonary disease. Additionally,16.2% of older adults experience multiple chronic conditions concurrently [18]. Although the attention to older adults is gradually increasing, the community will also organize some elderly activities, such as community health checkups, home care, square dances, and chess [19]. In this study, only 47.63% of community older adults with chronic diseases had regular physical examinations. Periodic physical examination is an essential measure to prevent diseases and promote health. In addition, it also provides an opportunity for health assessment, preventive health consultation, and the promotion of doctor-patient relationships. In some situations, such as cancer screening, periodic physical examination is critical [20]. The community should organize regular physical examinations for the elderly according to the needs of the older adults and dynamically monitor the changes in the elderly’s condition [21]. The study found that the health status of the elderly and male elderly is relatively low, so the community should pay more attention to them. Generally speaking, women live longer than men, but the gender gap is most significant in developed societies because women live about seven years longer than men on average [22]. Generally, men are more vulnerable to major life-threatening chronic diseases, including cancer, coronary heart disease, emphysema, cerebrovascular disease, liver cirrhosis, and so on [23].

A healthy population is a vital mark of a prosperous and strong nation. To comprehensively promote the construction of " Healthy China,“ China must deal with the coordination between health and economic and social development, and promote the improvement of health level must solve the contradiction between the supply of medical and health resources and services in the field of health and the health needs of residents [24]. Therefore, measuring the potential health needs of Chinese residents not only helps to understand the health needs of residents in the region but also has significant reference value for adjusting the allocation of medical and health resources from the regional level, improving the comprehensive utilization efficiency of medical services, and improving the health management system. This study used a cross-sectional survey to understand and analyze the health status and health needs of the elderly in the community. The cluster analysis method was used to explore the relationship between health needs items and investigate the commonness among health demand items, to provide a reference for the development of health management of the elderly with chronic diseases.

Theoretically, if the elderly have the exact health needs, they should receive the same health services [25]. However, the number of health services available to residents may vary due to factors such as income and medical insurance. The purpose of health management is to meet their own health needs. Accurately describing the current health needs of the elderly in China helps understand the quality of medical and health services and the allocation of medical resources in recent years. It can provide a decision-making basis for improving the health management system.

Health needs assessment is a systematic approach to ensure that the health service uses its resources to improve the population’s health status most efficiently [4]. In this study, the systematic clustering method was adopted to classify 39 indicators of community health needs, and the relationship between the indicators was clarified, which is helpful for community managers to provide services according to the classification and the preferred order of the large demand in each category, and improve the efficiency of community health intervention.

The survey results show that the health needs of the elderly in the community for various services are 1.3–69.7%, and the top three are: regularly organized physical examinations (69.7%), Day Care Center (67.4%), establishing a Medical Alert Systems (66.1%). The bottom three are: catheterization (1.3%), regular door-to-door service(2.0%), nasogastric feeding(2.1%). Regular health examination can prevent some chronic diseases and diagnose some early malignant diseases, which is of great value for the early diagnosis and treatment of diseases [26]. With the improvement of people’s living standards, we pay more attention to physical health in China. Functional disability and decreased body resistance are common in older adults. The phenomenon of population aging has led to a significant rise in the chronic disease rate compared to other human pathologies. Older adults are usually affected by > or = 2 chronic diseases concomitantly, mainly cardiovascular, pulmonary, and central nervous system diseases, metabolic disturbances and cancer [27]. These factors can potentially lead to the deterioration and debilitation of health status in the elderly. By physical examinations, you can reduce your likelihood of getting a chronic disease and improve your quality of life. By going to Day Care Center, older adults can overcome various age-related issues, including depression, loneliness, fatigue, and loss of brain function. Seniors can socialize, learn, move, and experience daily routines under the guidance of professional caregivers. As the aging population in China grows, Day Care Centers have now become a popular trend towards ensuring that the elderly are being well cared for and enjoy efficient and convenient services. One of the most significant benefits of installing an emergency call system is that it increases patient/resident safety. Falls, slips, and similar incidents are common risks in hospitals and senior living facilities. Table 3 results also show that first aid was an independent factor for health status.

The cluster analysis of health needs shows that 39 health needs items are divided into three categories, basic needs (24 items), health education (13 items), and first aid (2 items). Among the basic needs category, the top three with high need rates are regularly organized physical examinations, Day Care Centers, and increasing the construction of facilities and places for elderly fitness and entertainment activities in the community. In the health education category, the top three with high need rates are rehabilitation training and the use of rehabilitation training and education on the use of rehabilitation physiotherapy equipment, knowledge of medication safety, knowledge of dietary health, and understanding of common infectious disease prevention. The first aid category includes establishing Medical Alert Systems and emergency care.

Low SES leads to a heavier disease burden. For example, older adults experience more dental diseases and disabilities. This effect is global [28]. Overall, participating in social and medical insurance can help improve the health status of middle-aged and older adults to a certain extent and improve the self-evaluated health status of middle-aged and older adults, which can be understood as reducing the severity of the disease. Commercial medical insurance has no significant impact on older adults [29]. To realize the strategic decision of universal health, the Party Central Committee and the State Council have proposed to improve the medical security system, with basic medical insurance as the main body, and actively guide the construction of other forms of supplementary insurance and commercial medical insurance to form a multi-level and comprehensive perspective. China’s medical security system deepens and improves universal medical insurance [30].

Meet the basic service needs of the elderly in the community. Older adults in most communities have a greater demand for essential health services because they suffer from various chronic diseases. The National Basic Public Health Services (BPHS) is one of the priorities of the new health reform in China launched in 2009 [31]. Since 2009, the national BPHS project has been widely implemented in the primary health care (PHC) sectors, including community health centers (CHCs) and stations in urban areas, township hospital centers (THCs), and village clinics in rural areas of China [32]. These measures play an essential role in ensuring and improving the health condition of residents, and the fairness and availability of public health have greatly improved [33]. The community should establish a standardized archive system for the elderly and standardize the management of chronic diseases [34]. According to the needs of the elderly, organize regular physical examinations for the elderly, dynamically monitor the changes in the elderly’s condition, and increase the construction of community fitness and entertainment facilities and places for the elderly. Reasonably arrange community health service resources by the priority order of needs [35].

Health education is a process by which individuals and groups learn to behave in a way conducive to promoting, maintaining, or restoring health [36]. Health education increases individuals’ healthcare knowledge and enables them to understand their healthcare choices. World Health Organization (WHO) has emphasized the importance of health education to support health care needs and health promotion for older adults [37]. Strengthen health education to meet the needs of the elderly for medical care knowledge. Health education is a necessary tool to promote the health of older adults because it provides the ability to prevent and reduce diseases, enables the person to act in life transformation, and encourages self-care and the search for autonomy. However, it is essential to consider the older adults’ singularity to stimulate changes in individual behavior. Health education is critical to the community’s health needs [38]. The service should focus on health education to meet the needs of the elderly in the community for the use of rehabilitation training and rehabilitation physiotherapy equipment, drug safety knowledge, dietary health knowledge, common infectious disease prevention knowledge, etc. Through health education, to influence the behavior of the elderly to achieve three-level prevention [39]. Health education can take various forms, such as lectures on widespread science knowledge, street publicity, face-to-face health education, health education prescriptions, and holding publicity boards [40].

The occurrence of public health emergencies among the elderly in the community has become more frequent and more serious [41]. Attach importance to emergency first aid for the elderly in the community, and improve the emergency network system [42]. Emergency measures for emergencies should be strengthened, a Medical Alert Systems should be established, a transfer diagnosis system should be established, and the emergency network system should be improved. This study uses cluster analysis to classify 39 health needs items. It clarifies the relationship between the indicators, which is beneficial for community managers to provide services by the classification and the priority order of significant needs in each category and improve community health efficiency of the intervention.

The physical health of residents is not only an essential condition for economic and social operation, but also an inevitable requirement for promoting the overall development of individuals. Therefore, while increasing investment in medical and healthcare, our country should pay attention to whether the health needs of regional residents can be fully met and solve the contradiction between supply and demand in the healthcare field.

### Limitation

Some limitations of this study may apply, as we only involved a small proportion of participants from a local area in China and included only key socioeconomic factors in the regression models, which may not fully reflect the health status and needs of the entire older population in China. Future research should aim to address these limitations.

### Impact statement

Cluster analysis was used to explore the relationship among health needs items and analyze their commonalities, providing scientific reference for managing, evaluating, and intervening in the health of elderly individuals within the community.

## Conclusion

This study shows that 32.43% of the elderly suffer from a single chronic disease, and 67.47% suffer from two or more chronic diseases. The demand for home care among the elderly with chronic diseases in the community reaches 54.66% and 67.10%. Age, income and expenditure, medical expenses, health education, basic needs, and first aid were identified as influencing factors on health status. Health education remains the primary service item provided by community home care services. Therefore, the community should innovate its service model according to the home care needs of elderly individuals with chronic diseases. The community should also strengthen management of chronic diseases in the elderly, promote education and awareness related to these conditions, and provide complementary healthcare services based on individual health needs. This will improve overall health and quality of life for this population.

### Electronic supplementary material

Below is the link to the electronic supplementary material.

Supplementary Material 1

## Data Availability

The datasets generated and/or analysed during the current study are not publicly available to preserve anonymity of the respondents but are available from the corresponding author on reasonable request.
