# Supplementary material for: Classification of health needs: a cluster analysis of older adults in urban areas
Source: BMC Geriatr. 2023 Oct 9;23:638. doi: 10.1186/s12877-023-04333-y (PMC10563358; doi:10.1186/s12877-023-04333-y)
Supplement: Supplementary file 1 — Supplementary Material 1 [file 12877_2023_4333_MOESM1_ESM.docx]

**Supplementary Information**

Additional file 1.The SF-36 in English .

Additional file 2. Health needs Questionnaire in English.

**Additional file 1.** The SF-36 in English

1.In general, would you say your health is:

①Excellent ②Very good ③Good ④Fair ⑤Poor

2.Compared to one year ago, how would you rate your health in general now?

①Much better now than one year ago

②Somewhat better now than one year ago

③About the same

④Somewhat worse now than one year ago

⑤Much worse now than one year ago

3.The following items are about activities you might do during a typical day. Does your health now limit you in these activities? If so, how much?

(1) Vigorous activities, such as running, lifting heavy objects, participating in strenuous sports

①Yes, limited a lot ②Yes, limited a little ③No, not limited at all

(2) Moderate activities, such as moving tables, sweeping the floor, doing exercises, etc

①Yes, limited a lot ②Yes, limited a little ③No, not limited at all

(3) Lifting or carrying groceries,such as buying food, shopping, etc

①Yes, limited a lot ②Yes, limited a little ③No, not limited at all

(4) Climbing several flights of stairs

①Yes, limited a lot ②Yes, limited a little ③No, not limited at all

(5) Climbing one flight of stairs

①Yes, limited a lot ②Yes, limited a little ③No, not limited at all

(6) Bending, kneeling, or stooping

①Yes, limited a lot ②Yes, limited a little ③No, not limited at all

(7) Walking more than 1500 meters

①Yes, limited a lot ②Yes, limited a little ③No, not limited at all

(8) Walking for about 800 meters

①Yes, limited a lot ②Yes, limited a little ③No, not limited at all

(9) Walking for about 100 meters

①Yes, limited a lot ②Yes, limited a little ③No, not limited at all

(10) Bathing or dressing yourself

①Yes, limited a lot ②Yes, limited a little ③No, not limited at all

4.During the past 4 weeks, have you had any of the following problems with your work or other regular daily activities as a result of your physical health?

(1) Cut down the amount of time you spent on work or other activities

①Yes ②No

(2) Accomplished less than you would like

①Yes ②No

(3) Were limited in the kind of work or other activities

①Yes ②No

(4) Had difficulty performing the work or other activities (for example, it took extra effort)

①Yes ②No

5.During the past 4 weeks, have you had any of the following problems with your work or other regular daily activities as a result of any emotional problems (such as feeling depressed or anxious)?

(1)Cut down the amount of time you spent on work or other activities

①Yes ②No

(2)Accomplished less than you would like

①Yes ②No

(3) Didn't do work or other activities as carefully as usual

①Yes ②No

6.During the past 4 weeks, how much of the time has your physical health or emotional problems interfered with your social activities (like visiting with friends, relatives, etc.)?

①All of the time ②Most of the time ③Some of the time ④A little of the time

④None of the time

7.How much bodily pain have you had during the past 4 weeks?

①None ②Very mild ③Mild ④Severe ⑤ Very severe

8.During the past 4 weeks, how much did pain interfere with your normal work (including both work outside the home and housework)?

①Not at all ②A little bit ③Moderately ④Quite a bit ⑤Extremely

9.These questions are about how you feel and how things have been with you during the past 4 weeks. For each question, please give the one answer that comes closest to the way you have been feeling.

(1)Did you feel full of pep?

①All of the time ②Most of the time③A good bit of the time ④Some of the time ⑤A little of the time ⑥None of the time

(2)Have you been a very nervous person?

①All of the time ②Most of the time③A good bit of the time ④Some of the time ⑤A little of the time ⑥None of the time

(3)Have you felt so down in the dumps that nothing could cheer you up?

①All of the time ②Most of the time③A good bit of the time ④Some of the time ⑤A little of the time ⑥None of the time

(4) Have you felt calm and peaceful?

①All of the time ②Most of the time ③A good bit of the time ④Some of the time ⑤A little of the time ⑥None of the time

(5)Did you have a lot of energy?

①All of the time ②Most of the time③A good bit of the time ④Some of the time ⑤A little of the time ⑥None of the time

(6)Have you felt downhearted and blue?

①All of the time ②Most of the time ③A good bit of the time ④Some of the time ⑤A little of the time ⑥None of the time

(7)Did you feel worn out?

①All of the time ②Most of the time ③A good bit of the time ④Some of the time ⑤A little of the time ⑥None of the time

(8)Have you been a happy person?

①All of the time ②Most of the time ③A good bit of the time ④Some of the time ⑤A little of the time ⑥None of the time

(9)Did you feel tired?

①All of the time ②Most of the time ③A good bit of the time ④Some of the time ⑤A little of the time ⑥None of the time

10.Does your health limit your social activities (like visiting relatives and friends)?

①All of the time ②Most of the time ③A good bit of the time ④Some of the time ④A little of the time ⑥None of the time

11.How TRUE or FALSE is each of the following statements for you.

(1) I seem to get sick a little easier than other people

①Definitely true ②Mostly true ③Don't know ④Mostly false ⑤Definitely false

(2) I am as healthy as anybody I know

①Definitely true ②Mostly true ③Don't know ④Mostly false ⑤Definitely false

(3) I expect my health to get worse

①Definitely true ②Mostly true ③Don't know ④Mostly false ⑤Definitely false

(4) My health is excellent

①Definitely true ②Mostly true ③Don't know ④Mostly false ⑤Definitely false

**Additional file 2.** Health needs Questionnaire in English

| categories | Number | Health Needs Project | No need | Low need | Moderate need | High need |
| --- | --- | --- | --- | --- | --- | --- |
| I  Basic needs | 11 | All-day telephone consultation service |  |  |  |  |
|  | 15 | Day Care Centers |  |  |  |  |
|  | 16 | Oxygen inhalation |  |  |  |  |
|  | 17 | Increase the construction of facilities and places for elderly fitness and entertainment activities in the community |  |  |  |  |
|  | 18 | Regularly organized physical examinations |  |  |  |  |
|  | 19 | Blood glucose monitoring |  |  |  |  |
|  | 20 | Chronic disease case management / health card establishment |  |  |  |  |
|  | 21 | Electrocardiogram |  |  |  |  |
|  | 22 | Hospice care |  |  |  |  |
|  | 23 | Community geriatric care specialist clinic |  |  |  |  |
|  | 24 | Intramuscular and intravenous injection |  |  |  |  |
|  | 27 | Physical cooling |  |  |  |  |
|  | 28 | Wound dressing change washing |  |  |  |  |
|  | 29 | Family bed |  |  |  |  |
|  | 30 | Auxiliary expectoration |  |  |  |  |
|  | 31 | Help relieve pain |  |  |  |  |
|  | 32 | Oral health care |  |  |  |  |
|  | 33 | Specimen collection |  |  |  |  |
|  | 34 | Enema |  |  |  |  |
|  | 35 | Nasogastric feeding |  |  |  |  |
|  | 36 | Auxiliary defecation |  |  |  |  |
|  | 37 | Urinary incontinence bladder training guidance |  |  |  |  |
|  | 38 | Regular door-to-door service |  |  |  |  |
|  | 39 | Catheterization |  |  |  |  |
| II  Health education | 1 | Lecture on mental health |  |  |  |  |
|  | 2 | Rehabilitation exercise knowledge |  |  |  |  |
|  | 3 | Dietary health knowledge |  |  |  |  |
|  | 4 | Knowledge of medication safety |  |  |  |  |
|  | 5 | Knowledge of chronic health care |  |  |  |  |
|  | 6 | Knowledge of common infectious disease prevention |  |  |  |  |
|  | 7 | Catharsis on the use of rehabilitation training and rehabilitation physiotherapy instruments |  |  |  |  |
|  | 8 | Family first aid measures for cerebral hemorrhage |  |  |  |  |
|  | 9 | Knowledge of family rescue of hyperglycemia crisis or hypoglycemia coma caused by diabetes |  |  |  |  |
|  | 10 | First aid knowledge for the timely management of traumatic injuries in the fractured limb |  |  |  |  |
|  | 12 | Community Medical Care Welfare and Resource Introduction Center |  |  |  |  |
|  | 13 | First aid knowledge of poisoning |  |  |  |  |
|  | 14 | First aid measures for upper gastrointestinal bleeding |  |  |  |  |
| III  First aid | 25 | Emergency care for sudden illness |  |  |  |  |
|  | 26 | Establish a Medical Alert Systems |  |  |  |  |
